# Exploration of Community Health Workers’ views about in their role and support in Primary Health Care in Northern Cape, South Africa

**DOI:** 10.1007/s10900-019-00711-z

**Published:** 2019-08-07

**Authors:** Mokholelana Margaret Ramukumba

**Affiliations:** Department of Health Studies, University of South Africa, P O BOX 392, Pretoria, 0003 South Africa

**Keywords:** Community health workers, Community mobilisation, Community-based care, Ward-based primary health care outreach teams, Primary health care

## Abstract

Community health workers (CHWs) play a significant role in Primary health Care due to their proximity to households, communities and the health care system. Many studies focus on CHWs and the work they do. However, few have examined their experiences and identity and how that might influence how they view and perform their roles. The objectives of the study were to: Describe the role of CHWs in community-based health care in Northern Cape, Identify the perceived barriers and enablers to CHWs role performance, Explore CHWs views regarding the support from the communities and the formal healthcare system in Northern Cape. An exploratory qualitative design using focus groups was adopted. Forty-six (46) CHWs were purposively selected using the critical case sampling approach. Data were collected through three focus group interviews in three regions. Analysis followed the Graneheim & Lundman thematic analysis. Three themes emerged from data: perceived contribution to Primary Health Care, recognition of CHWs role, measures to improve working conditions. Findings showed that CHWs were engaged in various health and social care roles, they believed that they made a significant contribution to PHC, and that the health system persistently relied on their services. The enabler for finding meaning in their work was the positive community response and the good relations they had with the team leaders. The major barrier was the structure of the CHWs programme and the perceived lack of support by the government. The complex issues CHWs address in the community call for a review of their roles and workload as well as the support they receive from the formal healthcare system.

## Introduction

The Primary Health Care Re-engineering Model established in South Africa in 2010, puts strong emphasis on community-based care and includes social determinants of health in its approach. It integrates healthcare with health promotion and community participation [1] Within this model, Ward-based PHC Outreach Teams (WBPHCOTs) were established to deliver health promotion at ‘household and community level’ [2]. Each team comprises a professional nurse (team leader) linked to a health care facility, supervising six to ten CHWs. The main focus of the activities of teams is to strengthen health promotion, identify and support vulnerable individuals and families, based on the concept of a “healthy individual, a healthy family, a healthy community, and a healthy environment” [3, 4].

The National Department of Health (NDoH) sets targets for these teams, and these include household registrations, referrals, pregnant and postnatal women, under-five’s and promotion of treatment adherence for individuals with chronic illnesses [5]. The service is provided in collaboration with local health care facilities, other sectors and non-governmental organisations (NGOs) [1]. The District Health System primary role is to organize, manage and deliver PHC, including the CHWs programmes [6], skilled key health professionals and adequate resources are critical for the for the District to function optimally.

As in other low and middle-income countries, rural communities in South Africa face complex challenges such as, inadequate housing, poor access to basic health services, poverty, sanitation, water supply and other complex social problems [7]. Community health workers (CHWs) occupy a critical space in community-based health care because of their proximity to vulnerable individuals and communities, and the health care system [8]. They provide direct social and healthcare to families and the community [2], thus, playing a mediating role between the healthcare system and marginalized communities [9]. Therefore, they are increasingly regarded as nurses’ ‘extra pair of hands’ especially for maternal, new-born and child survival [10, 11]. However, this new level of workers needs to feel appreciated, supported to be able to make an impact on the health outcomes of the communities they serve.

Many studies focus on CHWs and the work they do. However, few have examined their experiences and identity and how that might influence how they view and perform their roles [12]. These perceptions may have a significant influence on their motivation and commitment to contribute meaningfully in primary health care. Their position within the healthcare system can either strengthen or break the healthcare system, if the programme is not managed effectively [13]. Considering CHWs significance in PHC, an exploration of their views about their role and support is critical.

These observations provided the opportunity to seek answers to the following research question:

*How do community health workers in Northern Cape perceive their role and support in Primary Health Care?*

The objectives of the study were to:

- Describe the role of CHWs in community-based health care in Northern Cape
- Identify the perceived barriers and enablers to CHWs role performance
- Explore CHWs views regarding the support from the communities and the formal healthcare system

## Methodology

An exploratory qualitative design using focus groups was adopted. The study was conducted in the Emthanjeni and Renosterberg municipalities situated within the Pixley ka Seme District in Northern Cape Province. These are two of the eight municipalities that make up the district [1].

Three groups totalling 46 community health workers were purposively selected using a critical case sampling to ensure a diverse representation of the different wards. This sampling technique is particularly useful in exploratory qualitative research where a single case (or small number of cases) can truly capture the phenomenon of interest [14]. The data were collected in three areas, named Region, A, B, and C (to protect their identities). An interview guide was developed based on study objectives and literature. The interviews lasted 50 to 60 min per group, they were audio-recorded and transcribed. Data was collected until data saturation was reached.

Qualitative content analysis followed the [15] approach. Data from the three groups were merged. The unit of analysis was the views of CHWs regarding who they were, what they did and how they felt about the support they received from communities and the department of health. The interviews were read several times to gain a sense of the participants’ perspectives. The text was divided into units of meaning that were condensed. The condensed units were abstracted and labelled with a code. The various codes were compared based on differences and similarities and sorted into eight subthemes. Subthemes were then grouped, resulting in three themes.

### Ethical Considerations

Participants received written information and signed an informed consent form. Permission was obtained for using a digital recorder. The study was approved by the Health Studies Research Ethics Committee, University of South Africa, the Northern Cape Province Department of Health and the sub-district Management team. Ethical measures such as respect for autonomy, beneficence, self-determination, non-maleficence and justice were observed and adhered to.

### Trustworthiness and Integrity of the Study

The trustworthiness of this study was enhanced through credibility, transferability, dependability, and confirmability. The study aimed to achieve these attributes by presenting the detailed description of the context of the study, the sampling process, and the analytic process and by illustrating the participants’ perspectives using direct quotations of their views. Member checking was used as a strategy to enhance clarity and meaning of data.

## Results

The interpretation of the results is presented under the headings of the three themes and sub-themes with quotations to show the emic perspective of CHWs. “An emic perspective attempts to capture participants' indigenous meanings of real-world events" [16]. Themes that emerged from data are: CHWs’ perceived contribution to PHC, Recognition of CHWs’ roles and measure to improve working conditions. These themes are integrated and feed into one another. Sub-themes are discussed under each theme.

### Theme: Perceived Contribution to Primary Health Care

#### Subtheme: Household Healthcare Activities

The community health workers indicated that their first mandate is to register households in allocated wards; the aim is to identify vulnerable individuals and families. They were aware of the PHC targets/indicators and emphasis on maternal and child health. They performed various health care activities such as screening, for pregnancy, chronic conditions, palliative care, wound care, registration of pregnancies, scheduled visits, checking immunization status of babies and if there were missing doses, they notified the team leader and referred the mother to the healthcare facility. To ensure adherence to medication, they traced “lost to follow up” cases and administered chronic medication, especially TB drugs. They believed that they were taking healthcare to the people.

We do visits, register members of the household, check how many children are under five, their immunisation status, and register all those who have problems. We have forms for children, adults, pregnancy, mental health and household tick sheet, we are basically taking the clinic to the communities

I often take medications to patients in their homes especially, the elderly, we visit them at home and check blood glucose and blood pressure. We work according to the indicators from the department of health. Once a week we discuss our targets with the team leaders to assess progress

When we enter households we not only focus on diseases, we offer support to the families, we check so many things like, road to health card, if the baby is gaining weight according to the chart, we give vitamin A and deworming pills. We DOT adults with TB, especially new cases in the first 2 weeks

We do wound dressings especially, for bedridden people, first we discuss the status of the wound with the sister or she might come and assess the wound and advise on what to use. When the wound is too big, we refer the patient to the clinic

#### Subtheme: Initiating Access Through Referrals

The CHWs assisted in initiating the smooth entry of the communities to healthcare services. They indicated that the household registrations allowed them to identify defaulters and lost to follow up cases, traced clients who are out of reach, such as those living on farms and completed the referral forms to the clinics. They referred needy clients to the clinic and other service providers, such as South African Social Security Agency (SASSA) for social grants, Social worker services, Home affairs, Municipality, as well as to the community centres that distribute food parcels to the poor. The screening tools enabled them to identify signs and symptoms of disease and refer accordingly. They received a “referral back” form from the facilities to complete the referral loop.

We found many children in the community who were not attending clinic and poor adults who were not getting grants so we referred them to SASSA and social workers so that they could receive their grants like other people

We help a lot with TB, our people used to report the disease when it was already at an advanced stage. When we enter households we do various screenings and when there is an indication we refer, we also encourage them to go for HIV testing. With tracing, we get addresses and trace them to faraway places like farms. Nurses at the clinics give us feedback about our referrals, we do a lot of referrals

#### Subtheme: Community Empowerment and Health Promotion

Participants claimed that the healthcare system has become reliant on their services and perceived their role as complimentary to the nurses’ in that they were their ‘eyes and ears’. They believed that their activities in the communities have reduced the queues and workload for nurses, because the majority of people are seen in their homes.

CHWs participate in various health promotions campaigns organized by the health facility, these involved screening for TB, cancer, male circumcision, physical activity for the elderly, adherence clubs and school health promotion. They accompany the professional nurse and assist with screening of Grade 1, 4 and 10 pupils, and made referrals to appropriate services. At schools they give medications as per protocol. According to the participants, the elderly are now getting better care because of home visits. They also render basic services at the health facility especially chronic care. They work with the nurse and the data capturer to record chronic cases.

I see myself as very valuable in PHC re-engineering, we love and support our communities. If things are not well in the family I know what to do. They do not have enough info so, I gather information for them because I am the government’s hand. At the clinic, we do blood pressure, blood glucose testing and check those on ARVs and keep records.

For the adherence clubs, we pair them with a buddy, go to the allocated place to get their medications to avoid long queues at the clinic. We also distribute condoms to shebeens (drinking venues), truck drivers, salons, tuckshops and garages. We use the re-engineering tool book 1 and 2 to give health talks.

When the sister comes from De Aar, we go to the schools and crèches to do the campaigns, we educate the kids, complete health forms, do MUAC and weight, administer Vit A and deworming pills

### Theme: Recognition of CHWs Roles

Participants expressed that both communities they serve and the department of health recognise their role of linking needy households with health and social service sectors. Two subthemes emerged from data: community response to CHWs interventions and support by the department of health.

#### Sub-theme: Community Response to CHWs Interventions

The majority of participants indicated that communities they serve show appreciation. They understood that their work was not easy, hence they motivated them to continue the good work by occasional tokens of appreciation. A few indicated that their approach was holistic, in addition, they provided emotional and spiritual support and that was well received by the families. They indicated that the communities took them as their saviors and usually consulted them anytime there was a need.

The community loves us, one day the church mama’s brought us 2 big cakes to encourage us

Community members who cannot afford to come to the clinic, are happy to get the services at home. Sometimes they do invite us to assist them. Some of them call us nurses, it feels good, but we tell them we are not

For me going into any household is not only for immunization or tracing defaulters, it is an opportunity to do spiritual work, and just listen to my clients. I found that I connect better with them that way

However, a few reported some level of hostility from young adults who are on TB and ARVs treatment. This negative response was attributed to alcohol abuse and the stigma associated with household visits.

Some people in the community do not understand this re-engineering because the home visits were initially for TB and HIV. They feel they are being stigmatized by the visits. The sister always advises me to persevere, they drink all the time and don’t take medication, it is not easy

#### Subtheme: Support by the Department of Health

The CHWs have good working relations with the nurses (team leaders), a good number acknowledged that the department of health is aware of their contribution, the majority expressed appreciation for the uniforms, and the prestige of being associated with the formal healthcare system, those small incentives encourage them to continue working.

One group in particular, appreciated how their team leaders support them and include them every morning in the review and mapping of the days’ activities. However, the majority claimed that the department is not doing enough. There is very little done to address their challenges such as high workload, exposure to health risks, inadequate resources, torn and old uniforms and no growth pathway. A few have Grade 12, yet when positions are advertised in the health facility, they are sidestepped. The torn uniform and shoes were particularly visible in Region A participants. The rest had good uniforms.

The main issues seemed to evolve around their 'unstable contracts’ and meagre stipends that are never guaranteed. They felt like they were in the periphery and only being used to extend the work of nurses.

We get stipend of R2500 per month from hospice, it is not enough. The money does not come regularly, we must call them and ask about the money and they will tell us they are also waiting for the province.

We have open contracts, our uniform is torn and old, we must buy trousers with our own money, they give us plastic shoes, when the sun is hot our feet hurt. We have BP machines but it takes a very long time for batteries to be replaced. We are exposed to unsafe environment sometimes, we go into household with MDR XDR patients and there are no masks or gloves when we check wounds. We cannot fight for our rights, we are always told that we are volunteers; we need to beg all the time. Even when we are sick, we are given one day and sometimes it is taken from your leave. We feel the department is just using us and nurses’ extensions

We are going for many years as volunteers and not moving upward. Department must take us seriously because now matric is a requirement. We started here still young but now we are old and when we want to study it's difficult. Maybe it will be better if after April (budget month) we are made permanent

### Theme: Measures to Improve Working Conditions

CHWs made several recommendations to support their activities in community-based health care delivery. The following subthemes emerged:

#### Subtheme: Remuneration and Incentives

CHWs believed that they have enough experience to be formally integrated into the healthcare system and given permanent positions. Much as they appreciated the role of NGOs in the recruitment and remuneration, they felt that the NGO is also helpless, the department has more authority to make strategic decisions regarding their employment status. They indicated that a permanent position with a salary that will allow them to register with Unemployment Insurance Funds scheme and leave benefits. Since the majority of CHWs are women, they wanted reassurance of a better quality of life and a future for their children.

I have been working for many years getting a stipend instead of a salary. What I need is that after my death my children must be able to get something. I don’t even contribute to UIF because I’m not registered. The leave should be revised, so that we have a reasonable sick leave

Those of us above 35 need permanent jobs, NGOs also suffer because the money for our stipends comes from the government. We need to be well supported, the work allocated to us is unreasonable, it should be reduced and be better structured so that we can provide quality work, currently, we are all over the place doing this and that

#### Subtheme: Health and Safety Issues

Participants suggested several ways their health and safety could be enhanced. In this region, their main concern was health risks as they visited patients with highly infectious diseases, they suggested the department to provide them with adequate protective equipment and provide policies regarding their health in case they contract any of the diseases. The security could also be improved, for them to feel safe when they enter households. In addition, they wanted to have an input in the drawing up of their contracts.

There must be a protection plan for us, like, we could have free physical examination twice a year and treatment as needed, we are teaching clients about disease prevention, we need it too. There’s a lot of XDR and MDR in our communities, we need good masks

We must have inputs and outputs into the contract. We go into the community and anything can happen. So we need benefits like accident cover and sick leave needs to be revised

#### Subtheme: Opportunities for professional Growth

The majority of CHWs suggested some improvements in recruitment and training. They believed this would increase the likelihood of being absorbed into the health system. They would prefer the department rather than the NGOs, to do recruitments. They are content with the training offered by the department. However, they would prefer regular and formal updates that would lead to formal recognition. This mainly came from those with ambitions for further growth. The majority of them had only phase 1 training, there were a few, especially from the social development sector who have had no training. The older CHWs recommended the age criteria to be reviewed as the current requirement disadvantages them.

Currently you pass the courses but remain in the same level, there must be some formal certificates that will enable us to go into the next level. It would be better if the department advertises the CHWs posts and we can apply directly

They tell us they want 18–35 yet, we have been working here for years. This is hurting I have been working here for more than 5 years without matric but doing the work of professionals

#### Subtheme: Improvement in Data Management

The participants recognised that the healthcare facilities rely on their inputs. Therefore, they had to manage data effectively. Currently, CHWs felt that data recording is tedious and there are too many forms to complete. The storage of data is not ideal and the security of data is not guaranteed. All participants lamented the termination of the mobile health initiative that enabled them to capture data fast and effortlessly and visit more household per day.

We still have our tablets, but they were stopped because of some technical problems. We do not know when we will use them again, it was good because we were able to register at least 10 households per day and do our referrals with ease. Now when I visit households, I have to carry maternity records, individual records, tick sheets, TB records etc. the tablet had all the information

When coming to reporting at the end of the month it was much easy (sic). With paper, we experience problems. The papers are in the files that we keep in our room, too much paper and reports can be untidy. The government must bring back smaller tablets that can fit into our bags

## Discussion

The study presented the voices of CHWs regarding their roles in Primary Health Care especially, at household and community level. CHWs demonstrated awareness of their importance to the department of health and the communities they served. They articulated their roles in ways that were consistent with the PHC Re-engineering principles. Their roles in maternal child health interventions, integrated approaches to chronic conditions, referrals and health promotion are key themes in PHC. They were aware of the targets they needed to achieve, expectations in relation to their responsibilities, scope of practice and reporting requirements. They believed that they were in a better position to detect diseases early and link the needy to the relevant services. The deployment of CHWs to increase access to health care, acceptability, relevance and to support health care provision has been documented in several studies [3, 5, 8 ].

The results suggests that CHWs regarded themselves as agents of change, in that the household registrations allowed them to identify vulnerable individuals and take appropriate action, thus, improving access to care. Meaning that they effected noticeable change in the lives of the communities [17], agree that the household profiling enables CHWs to identify vulnerability. They perform various screening activities which would normally be performed by nurses, hence, they believed that their interventions have alleviated some workload on health facilities, as there were lesser queues at the facilities. These findings were not verified with the facilities. The role of linking the communities and the formal healthcare system was evident through referrals to various entities such as SASSA, among others. The referral procedures seemed to be well established, in that there were back referrals from either the team leaders or the facilities.

They have also empowered the families and individuals to take control of their own health by providing health education on various topics such as male circumcision, immunisations, nutrition and adherence. These are key health promotion issues, in view of the disease burden in South Africa [18–20] acknowledge their importance in promoting a healthy lifestyle and providing educational information to communities. They believed that this role has enhanced their status in the community in that the majority of families and communities responded positively to their interventions. The gratitude they received gave value and meaning to their work. Being associated with medical knowledge can be empowering and a source of inspiration for the CHWs [21]. Therefore, CHWs need to be adequately supported and their work acknowledged [6] confirm that the generalist roles of CHWs is a valuable and potential intervention for the re-engineering of the primary health care system in South Africa, because it creates a model of integrated care across different levels of the health care system. However, this study found that this generalist role complex and heavy for them, more so, the quality of work they provide has not been assessed.

The working relations with the team leaders was perceived as good, this is reflective of the level of support team leaders (professional nurses) provided. However, all participants claimed that the support from the health care system was less desirable. Their main challenge was the high workload, lack of resources and open contracts that generate anxieties over their future. This finding is consistent with [22] study that revealed that for CHWs to be able to function effectively and to be able to empower the communities they serve, it is essential that they themselves be or feel empowered. Training opportunities for growth and irregular stipends were cited as barriers to self-actualisation in their work. The Community health workers come from impoverished families and communities [8], depriving them of basic necessities, yet entrusting them with the health of communities is a threat to the core principles enshrined in PHC. The healthcare system needs to invest in building CHWs' skills and support them as valued members of the health Team [8]. Adequate training and support are crucial in empowering the CHWs [19].

The recommendations CHWs made were mainly to improve their working conditions. First and foremost, they suggested that the workload needs to be reviewed and adjusted. This begs the question: what is the most reasonable or practical workload CHWs can take? Various factors would need to be considered, also, caution needs to be exercised to prevent demotivation and burnout. There are complex reasons why CHWs seek more compensation: poverty and a need to provide for their families [8]. The suggestion to participate in drawing up their contracts is well founded and supported in literature, CHWs need to be engaged in the policy process, help shape new standards for CHW programs based on rooting out social and economic inequities, and develop appropriate solutions to complex CHW policy problems [8, 23].

Their concerns about their health, security and safety are well founded, crime is a reality in South Africa as well as various forms of resistant TB prevalent in the communities. They showed awareness of the importance of quality of health data. Hence, they wanted the tablet initiative to resume, with adequate and accessible technical support [6], argue that the healthcare system is grappling with its own fundamental challenges. District teams are also facing complex financial and human resources challenges [24]. This would invariably impact the distribution of resources and effective integration of CHWs into the formal healthcare system.

### Limitations

This study is limited to the two sub-districts in one district. The data are from a small number of CHWs. As such, data may not represent the perceptions of CHWs in other areas in the province. Furthermore, the perceived effectiveness or quality of their interventions were not collected in the current study and as such the quality of their practices remain unknown.

## Conclusion

Findings show that CHWs believed that they were making a meaningful and significant contribution to health promotion and the re-engineered PHC approach. They had diverse responsibilities, and most of their activities seemed to involve home-based care, palliative care, support, health, social care and referrals. The complex issues CHWs address in the community call for a review of their roles and workload as well as the quality of work they provide. Their scope of work needs to be clarified and be congruent with the training provided. Their participation in healthcare facilities could create a dilemma regarding reporting processes. The involvement of other healthcare professionals at the facilities would require increased awareness or knowledge of their roles and responsibilities. At the same time, it could create a pathway for effective integration into the system. This observation calls for greater policy directives and clearer definition of roles of all stakeholders are primary care level.

CHWs perception of support from the government is a threat to the sustainability of this programme. Currently, they felt as if they were at the margins of the health care system, their needs and challenges seemed to have not been adequately addressed. It is evident from findings that urgent policy directives regarding their position, workload, training, resources such as meeting rooms and proper storage of files, and health and safety issues are required. In addition, the role of NGOs need to be clarified, as one of the recommendations was that recruitments should be handled by the department.

The positive response by the communities could be an opportunity or a vehicle for the government to involve communities in the recruitment of CHWs. In the absence of their mobile devices, documentation of CHWs data needs to be reviewed, the current data collection tools are viewed as tedious and a barrier to good health information management. This study assumes that adequate support for CHWs would enable them to perform effectively in their roles and better respond to the community and family needs. The health system policy could sustain their commitment and motivation to serve their communities by addressing issues such as their desire for knowledge, hope for permanency and financial incentives.
